# Advancement and emerging challenges in electric-field assisted manufacturing: a review

**DOI:** 10.1007/s00170-025-16499-3

**Published:** 2025-11-04

**Authors:** Seikh Mustafa Kamal, Lorenzo Zani, Ahmad Abdul Kadir, Konstantinos P. Baxevanakis, Anish Roy

**Affiliations:** 1 Department of Mechanical Engineering, Tezpur University, Tezpur, 784028 Assam, India; 2 Wolfson School of Mechanical, Electrical and Manufacturing Engineering, Loughborough University, Loughborough, UK

**Keywords:** Electric-field assisted manufacturing, Hybrid manufacturing, Electroplasticity, Electrically assisted machining

## Abstract

Electric-field assisted (EA) manufacturing is a promising hybrid manufacturing technique, offering significant advantages over conventional manufacturing methods. Extensive experimental and numerical studies have demonstrated that the application of electric current reduces flow stress in metals and alloys, thereby improving their manufacturability. This enhancement is attributed to the synergistic effects of electroplasticity and Joule heating, both induced by the applied current during processing. Several key manufacturing processes have garnered substantial interest from the research community for their potential enhancement through electric fields. Here, we present a comprehensive review of recent developments in EA manufacturing over the last decade. The findings of various researchers investigating different EA manufacturing processes are discussed, accompanied by detailed tables summarizing the materials and electric current parameters employed in each process.

## Introduction

The focus of the modern-day manufacturing industry is to produce parts through efficient manufacturing processes in terms of time, cost, and energy consumed during its manufacture without compromising the structural integrity of the manufactured component. Hybrid manufacturing techniques have been proven as a viable alternative to conventional manufacturing processes to address these challenges [1–4]. In recent times, electric-field assisted (EA) manufacturing processes have emerged as one of the promising techniques to improve the manufacturability of different hard-to-process materials. In EA manufacturing processes, an electric field is applied to the parts to be processed in the form of either continuous or pulsed direct current (DC). The pulsed current may be applied with different waveforms, such as sinusoidal, square or saw-tooth, with varying pulse frequency and amplitudes. The current imposed in an EA manufacturing process is usually measured in terms of its current density (in A/mm^2^), which measures the amount of current flowing through the unit cross-sectional area of the part or component under manufacture.

The effects of using an electric field during material processing were recognized in some of the earliest works by Troitskii [5, 6], Okazaki et al. [7], and Conrad et al. [8]. It was reported in the literature that the application of an electric field favorably influences the material and microstructural properties of various hard-to-deform materials such as titanium, aluminum, and magnesium alloys during their deformation. For example, the application of either continuous or pulsed DC in a uniaxial tensile test causes a reduction in flow stress and fracture strain [9–14]. Similarly, an electrically assisted compression test leads to a reduction in compressive flow stress, a decrease in Young’s modulus, enhancement in formability, and elimination of microcrack formation and propagation [15–17]. Apart from tension and compression properties, the use of an electric field in material deformation also influences grain size [18–21], recrystallization [18, 22–24], creep rate and diffusion [24, 25], and fatigue [26, 27].

Mainly driven by the classical work of Troitskii [5, 6], in the early twentieth century, many researchers focused on the development of EA manufacturing processes initially associated with metal forming processes, viz., rolling, forging, wire drawing, and sheet metal forming [28–32]. Some of the recent works on EA forming were reported in [33–36]. Subsequently, research also sought to innovate other EA processes associated with machining [37–39], metal joining [40–43], and other processes, such as EA surface treatment processes [44, 45]. In general, it was reported that the application of electric current induces a reduction in forming forces and springback in metal forming, a reduction in cutting force and enhanced surface finish in machining/metal cutting, an increase in bond strength in metal joining, enhanced ductility and refinement of grain size as compared to the conventional manufacturing processes. These benefits are generally attributed to two key mechanisms: thermal and athermal effects. The thermal contribution arises from *Joule heating*, which causes a localized temperature rise within the material, thereby reducing its resistance to deformation and resulting in lower flow stress and hardness. In parallel, the athermal *electroplastic effect*, primarily associated with enhanced dislocation mobility under the influence of an electric current, further improves deformability. This phenomenon is often linked to electron-dislocation interactions, commonly referred to as the electron wind force, which facilitates easier dislocation movement and reduces yield strength [6, 17, 46–51]. Together, these thermal and athermal effects contribute to increased formability and material softening, ultimately lowering the mechanical energy required during processing. Experimental studies support the foregoing mechanisms; for instance, tensile tests on Ti-based alloys with pulsed current application have shown up to a 30% reduction in flow stress, while Al and Mg alloys exhibit decreased hardness and refined microstructures under similar conditions [9, 10, 14, 18, 19]. However, it is important to note that the electroplastic response varies between materials, indicating a strong material dependency [52]. Among the proposed mechanisms, the electron wind effect is frequently cited as a driving factor for the observed electroplasticity [6, 53]. A comprehensive overview of the electroplasticity mechanism can be found in a review paper by Ruszkiewicz et al. [54].

Although the foregoing literature supports the athermal electroplastic effect in electric field-induced deformation in metals, a few works have contradicted this effect [55–57], leaving the topic debatable. A similar effect, often referred to as magnetoplasticity, induced by a magnetic field due to the flow of electric current during plastic deformation of materials, was also recognized [58] and applied to develop hybrid manufacturing processes [59, 60]. The magnetoplasticity in metals is attributed to the strong magnetic field capable of increasing the electron component of the viscous deceleration of dislocations and thus decreasing the plasticity.

An extensive review of the work done on EA manufacturing processes until early 2014 was presented in [61]. Although significant progress has been made in the further development of various manufacturing processes to date, no comprehensive review of EA manufacturing processes providing the current state-of-the-art on the subject is available in the literature. Therefore, a comprehensive review of all known EA manufacturing processes is carried out, covering the research works conducted in the last decade. The purpose of this review is to provide the current understanding of electroplasticity in various manufacturing applications and discuss its current and future challenges.

## Current state-of-the-art on electric field assisted manufacturing processes

In the last decade, considerable efforts were made to understand the electroplasticity effect in manufacturing through experimentation and modelling. In this section, observations of various EA manufacturing processes on different materials are reviewed, and future challenges are discussed in Section 2.3.5.

### EA machining processes

The modern-day machining processes aim at enhancing the machinability of materials at the cost of reduced power consumption or removing materials from hard-to-machine materials with ease, without compromising the surface integrity and other associated mechanical properties of the material. Researchers have found EA machining processes as one of the potential alternative hybrid manufacturing procedures to meet the above requirements. The EA machining processes that have primarily been investigated are EA turning and EA drilling processes. However, EA milling has received very limited attention to date.

#### EA turning

**Fig. 1:**
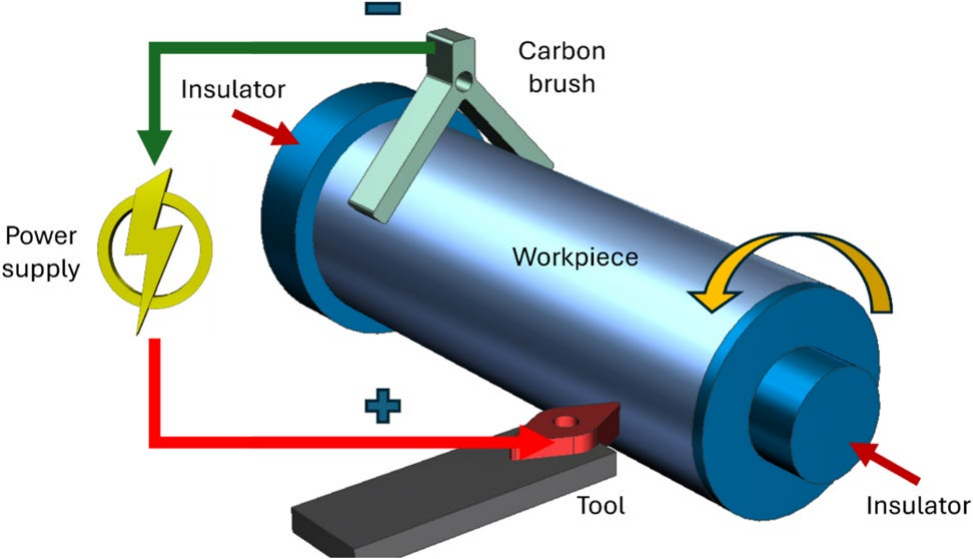
Schematic of EA turning process

A typical EA turning process is schematically shown in Fig. 1. All studies on EA turning use direct current (DC), in either pulsed or continuous form, to achieve the added advantages of electroplasticity during material removal. Egea et al. [62] carried out an experimental study on the effect of electroplasticity in turning of different steel grades, i.e., SAE1020, SAE1045, and SAE4140, using an in-house electropulse generator. The machining was performed employing a current of 90 A for a pulse duration of 50–200 μm. They observed improved machinability, with a reduction in surface roughness ranging from 6 to 40%, and a decrease in surface hardness between 6 and 16% compared to conventional turning. Additionally, the presence of electropulses led to lower power consumption by as much as 105.4 W and a 25% reduction in specific cutting energy, especially under high-frequency, long-pulse conditions.

To study the influence of currents in EA machining, Ulutan et al. [63] applied different currents ranging from 50 to 600 A at the tool-workpiece interface in the turning of Ti-6Al-4V and Inconel 738 alloy rods using a Darrah 4 kA continuous DC power supply. They found that electroplasticity reduced machining forces once the current exceeded 50–100 A, remaining effective up to 300–400 A. Beyond this range, machining forces increased, indicating deteriorated performance. For Inconel 738, machining forces decreased by 70% at 300 A compared to the no-current condition, but increased by 50% when the current was raised to 400 A. Similarly, for Ti-6Al-4V, machining forces were reduced by over 20% at 400 A but increased by more than 40% at 600 A compared to the force without current.

Wang et al. [64] carried out EA turning of AISI 304 stainless steel, applying current densities ranging from 7.43 to 14.45 A/mm^2^ using an electropulse generator of a maximum capacity of 5 kA with a pulse width of 60 μs. They observed a maximum 23.6% reduction in main cutting force and a 22.6% decrease in microhardness at a root mean square (RMS) current density of 1.60 A/mm^2^ and 600 Hz compared to conventional turning. Beyond this current, force reduction slowed, and microhardness increased, likely due to surface oxidation caused by high temperature and strong current. Axial surface roughness decreased significantly, reaching a minimum Ra of 1.32 µm at 1.28 A/mm^2^ and 700 Hz, representing a 61.7% improvement over the 3.45 µm in traditional turning. Further, electropulsing aided in the reduction of tool-workpiece friction and improvement in the lubrication properties. A similar effect of electropulsing was also observed by Sun et al. [39] and Chen et al. [65] in EA machining of GH4169 superalloy and quenched and tempered 45 steel rods, respectively, under suitable pulsed current parameters. Lou and Wu [66] reported a decrease in cutting forces with the formation of continuous chips and a better surface finish while performing ultra-precision machining of electropulsing-treated Ti-6Al-4V alloy. Due to electropulsing, the yield stress and hardness of the alloy were reduced, and the maximum temperature was raised to 600 °C. Their observations were based on finite element simulations supported by experiments.

An electropulse generator of 9 kA capacity with current densities 3.6–7.29 A/mm^2^ for a pulse duration of 75 μs was used by Xu et al. [38] in the EA turning of AISI 5120 cementation steel. They reported a reduction in cutting force by 24.3% as compared to the conventional turning process, along with a reduction in tool wear and improvements in the quality of the machined surface. In a recent study, Guan et al. [67] experimentally investigated the EA turning of TC27 titanium alloy using two types of cutting tools, TiAlN-coated cemented carbide insert and uncoated carbide insert, and compared the results with conventional turning. The improvements in comparison to conventional turning were demonstrated by a reduction of more than 20% in cutting force and of about 30% in the surface roughness of the turned surface with a root mean square of current density 0.9 A/mm^2^. The EA machining performance was found to be slightly superior using the TiAlN-coated cemented carbide inserts than the uncoated carbide inserts in terms of cutting force, surface roughness, work hardening, and resistance to wear. The optimized electrical pulse parameters reported in the study were as follows: pulse frequency of 600 Hz, an amplitude current density of 0.57 A/mm^2^, and a pulse length of 100 μs.

Recently, Zhao et al. [68] experimentally investigated EA dry turning of W93NiFe alloy under different pulse electrical current parameters on machining performance, especially on the surface roughness, surface defects, tool wear, and chip formation, and compared this with the conventional dry turning process. The authors observed a gradual decrease in the surface roughness value with an increase in pulse voltage, reaching the minimum value at the pulse voltage of 80 V. A maximum reduction of 38.94% in surface roughness in EA turning was achieved when compared to conventional dry turning. EA turning reduced machined surface defects and tool wear, albeit with the formation of built-up edge (BUE) at the tool tip. During EA machining, longer transverse lengths of ribbon chips with smaller chip curl radii were formed. The details of the EA machining parameters, along with the cutting tool and workpiece used in all the preceding studies, are summarized in Table 1.

**Table 1:** EA turning

Process parameters	Current	Specimen and cutting tool	Reference
Spindle speed: 460 rpm Feed rate: 0.046, 0.127, 0.254, 0.356 mm/rev Depth of cut: 0.25 mm	Pulsed DC Current: 90 A Pulse duration: 50–200 μs Frequency: 100–300 Hz	SAE1020, SAE1045, SAE4140 steel rods *ϕ*25 × 190 mm TNMG-16 tool	Egea et al. [62]
Spindle speed: 350 rpm Cutting speed: ~ 42 m/min Feed rate: 0.04, 0.06 mm/rev	Continuous DC Current: 50, 100, 200, 300, 400, 600 A	Ti-6Al-4V and Inconel 738 rods *ϕ*38 × 4 mm	Ulutan et al. [63]
Cutting Speed: 40 m/min Feed rate: 0.2 mm/rev Depth of cut: 0.6 mm	Pulsed DC Maximum current density: 7.43, 9.15, 12.09, 14.45 A Frequency: 500, 600, 700 Hz Pulse duration: 60 μs	AISI S304 steel rods *ϕ*12.6 × 150 mm C-type YG6X cemented carbide tool	Wang et al. [64]
Cutting speed: 26.4 m/min Feed rate: 0.2 mm/rev Depth of cut: 0.1 mm	Pulsed DC Maximum current density: 0.34, 0.51, 0.64, 0.8 A/mm^2^ Frequency: 300, 400, 500 Hz Pulse duration: 90 μs	GH4169 superalloy rods *ϕ*21 × 120 mm YG6X tool	Sun et al [39]
Spindle speed: 900 rev/min Cutting speed: 45.2 m/min Feed rate: 0.2 mm/rev Depth of cut: 0.5 mm	Pulsed DC Maximum current density: 0.39, 8.98, 9.22, 10.12, 10.96, 12.63, 13.14, 14.29 Frequency: 300, 400 Hz	Quenched and tempered 45 steel rods *ϕ*16 × 200 mm	Chen et al. [65]
Spindle speed: 2000 rpm Cutting speed: 45.2 m/min Feed rate: 15 mm/min Depth of cut: 3 μm Tool nose radius: 0.772 mm Tool rake angle: 0º Front clearance: 10º Cutting edge radius: 0.75 μm	Pulsed DC Current density: 2.286 × 10^5^ A/cm^2^ Frequency: 500 Hz Voltage: 2.64 V	Cylindrical Ti-6Al-4V alloy sample *ϕ*14 × 120 mm Natural diamond tool	Lou and Wu [66]
Spindle speed: 400 rev/min Cutting speed: 21.2 m/min Feed rate: 0.1 mm/rev Depth of cut: 0.3 mm	Pulsed DC Maximum current density: 7.43, 9.15, 12.09, 14.45 A Frequency: 500, 600, 700 Hz Pulse duration: 75 μs	AISI 5120 cementation steel rods *ϕ*17.0 × 170 mm NX2525 tool	Xu et al. [38]
Cutting speed: 40 m/min Feed rate: 0.1 mm/rev Depth of cut: 1 mm	Pulsed DC Current densities: 0.67, 0.89, 1.42 A/mm^2^ Frequency: 500, 600, 700 Hz Lasting time of the current per circle: 100 μs	Ti5A14Mo6V2Nb1Fe (TC27) alloy *ϕ*16 mm, length: 180 mm Tools: TiAlN-coated cemented carbide insert and uncoated carbide insert	Guan et al. [67]
Spindle speed: 300 rev/min Feed rate: 0.08 mm/rev Back cut: 0.08 mm Cutting length of each section: 10 mm	Pulsed DC Maximum voltage: 130 V Maximum frequency: 800 Hz Pulse width: 50–200 μs	W93NiFe alloy *ϕ*30 mm, Length: 270 mm Tool: CNGG 12 04 04-SGF blades made of H13A with a tip arc radius of 0.4 mm and a cutting edge length of 8.5 mm	Zhao et al. [68]

#### EA drilling

An in-house EA drilling process was designed and developed by Hameed et al. [37] using a DC micropulse generator with a constant current intensity of 120 A. The process was exemplified by drilling 7075 Al and 1045 carbon steel at 140 A current for a pulse duration of 250 μs. The authors reported a reduction in cutting energy due to electropulsing, ranging from 17 to 27% in the cutting of 7075 Al and 10–17% in the cutting of 1045 carbon steel, as compared to the ordinary drilling process. The EA drilling of automotive grade Usibor 1500 boron steel was carried out by Karumatt et al. [69] to study the influence of current on the axial force experienced by the tool during drilling. Continuous DC currents varying from 0 to 500 A to the workpiece through a tungsten carbide-tipped steel drill bit using a 4 kA Darrah power supply were imposed. The load-controlled current was applied upon exceeding a threshold load of 220 N to circumvent arcing during the process. The electroplastic effect was prominent at a current of 360 A without tool chipping and with a slight reduction in average axial force as compared to the cases at 0 A and 180 A. Although at currents higher than 360 A, a slight increase in average axial force on the tool was observed, the increased chip continuity at higher currents tends to increase the tool life.

The effect of electric current in EA drilling of mild 1008CR steel and high-strength PHS1500 steel through experimentation and modelling was investigated by Ruszkiewicz et al. [70]. A detailed design of experiment on 1008CR steel was carried out to determine the effect of the primary process parameters. It was observed that electric current can reduce cutting forces at the cost of an increased workpiece temperature. Studies in PHS1500 reveal that force can be reduced by 50% at high feed rates without observing catastrophic tool failure for up to ten cuts at 600 A, while tool failure occurs in only a single cut for 0 A (conventional machining). The authors also proposed an electroplastic drilling model to predict cutting forces and temperatures in the presence of electric current. An overview of the process parameters used in the above experimental studies of the EA drilling process is summarized in Table 2.

**Table 2:** EA drilling

Process parameters	Current	Specimen and cutting tool	Reference
Spindle speed: 1050 rpm Feed velocity: 0.2 to 0.4 mm/s	Pulsed DC Current: 140 A Pulse duration: 250 μs Avg. output power: 300 W Frequency: 300 Hz	7075 Al, 1045 carbon steel Tool: twist drill high-speed steel (HSS), point angle 116–120°º and helix angle 20–30°	Hameed et al. [37]
Spindle speed: 560 rpm Feed rate: 25.4 mm/min	Continuous DC Current: 0, 180, 360, 500 A	Usibor 1500 boron steel Drill bit: Tungsten carbide-tipped steel, 6.35 mm diameter, 117° point angle	Karumatt et al. [69]
**For 1008CR steel** Spindle speed: 350, 560 rpm Feed rate: 50.8, 101.6 mm/min **For PH1500 steel** Spindle speed: 560 rpm Feed rate: 12.7, 25.4 mm/min	Continuous DC Current: 0, 150, 300 A (for 1008CR steel) 0, 300, 600 A (for PH1500 steel)	1008CR steel, PHS1500 steel Drill bits: Black oxide steel bits with a point angle of 135° (for drilling 1008CR) Tungsten carbide-tipped steel bits with a point angle of 117° (for drilling PHS1500)	Ruszkiewicz et al. [70]

#### EA milling

It is important to highlight that electric-field assisted milling remains one of the least explored areas within EA manufacturing. The limited research attention to date may be attributed to the inherent complexities in the milling process, involving intermittent cutting, multi-axis tool paths, and varying engagement conditions due to the rotating cutter and dynamic tool-workpiece interaction. These factors pose challenges in maintaining a stable and consistent electrical connection during the milling operation. The integration of electrical currents into such a dynamic environment complicates the system design of electrically assisted milling, particularly in ensuring effective current transmission without compromising tool integrity or machining stability. Nevertheless, emerging studies started to explore this process. For example, recently Fujian et al. [71] investigated electric pulse-assisted helical hole milling of TC11 titanium alloy using the following pulse current parameters: duty cycle of 20–80%, current density of 0.1–0.25 A/mm^2^, and pulse frequency of 100–400 Hz. The cutting parameters for the milling operation were taken as follows: spindle speed (milling cutter rotation) 1800 rpm, feed per revolution per tooth 0.025 mm/z, and axial depth of cut 0.2 mm. Their findings demonstrated that electric pulse-assisted milling can significantly enhance machining performance, showing a 23.1% reduction in surface roughness compared to conventional milling. Additionally, it was reported that the friction coefficient of the machined hole decreased by approximately 50%, indicating improved tribological performance and surface integrity. These promising results suggest that, while electric-field-assisted milling presents technical challenges, it holds considerable potential for improving machining outcomes, particularly for difficult-to-machine materials.

### EA joining processes

To achieve a certain edge over conventional joining processes, such as to achieve efficient joining between two materials or to increase bond strength, electrically assisted welding processes have emerged as a promising technique. There have been quite a number of experimental works and theoretical modelling on EA joining, mainly associated with solid-state pressure welding and friction stir welding. The proposed EA joining processes could find their potential applications in joining two dissimilar or difficult-to-join materials in industries where conventional joining processes are not efficient or suitable.

#### EA pressure joining

**Fig. 2:**
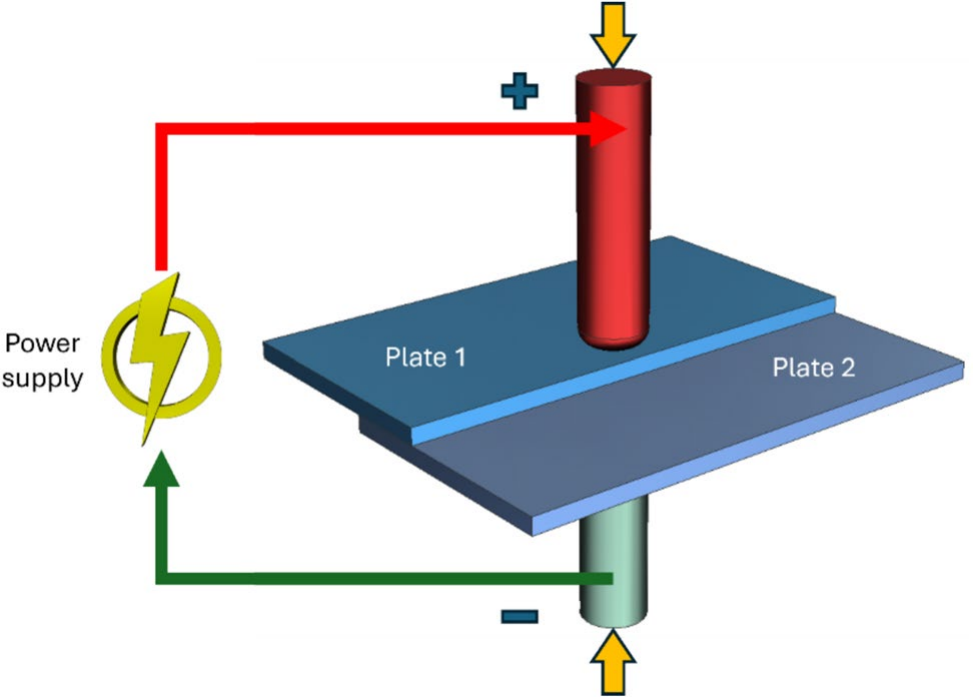
A lap joint undergoing EA pressure welding

The principle of EA pressure joining is based on the application of direct electric current at the joint between the two materials under continuous axial compressive pressure. The application of electric current stimulates the plastic flow of the base material to facilitate enhanced solid-state bonding performance. A typical lap joint undergoing EA pressure joining is schematically shown in Fig. 2.

The various experimental investigations on the EA pressure joining process of different, similar, or dissimilar materials are summarized in Table 3. Most of the studies focused on the joining of two dissimilar materials. Peng et al. [72] proposed an EA pressure welding process for the dissimilar joining of thin metallic sheets of stainless steel and brass. They developed an experimental setup to apply continuous DC at the joint by employing a power source, MYS 1600–05, with a maximum power output of 1 kW. The welding force was provided by using a KQL test machine of 100 kN capacity at a speed of 0.6 mm/min. The authors carried out the EA pressure joining experiments under different process parameters, applying current densities ranging from 6.7 to 20 A/mm^2^. Assessing the bond strengths at different current densities, they reported that the bond strength increased up to 36% with increasing current density from 6.7 to 20 A/mm^2^. The authors also developed an analytical model to validate the experimentally obtained bond strengths based on the film theory, incorporating the virgin metal’s and the contaminant layer’s deformation.

**Table 3:** EA pressure joining

Process parameters	Current	Specimen	Reference
Preload 100 N Compression rate: 0.6 mm/min Compression force: 4.3–60 kN	Continuous DC Current density: 5, 6.7, 10, 13.3, 20, 25 A/mm^2^	CuZn40 and SS 316 foils Thicknesses: 0.1 and 0.15 mm	Peng et al. [72]
Compression rate: 24 mm/min Max displacement: 6 mm Compression force: 27–38 kN	Continuous and pulsed DC Current density: 32, 19 A/mm^2^ Current duration: 5, 0.75 s	SUS316L bars *ϕ*10 × 15 mm, *ϕ*10 × 13 mm, *ϕ*10 × 4 mm	Hong et al. [73]
Preload 100 N Compression rate: 12 mm/min Compression force: 2.4–10 kN	Continuous DC Current: 1.2, 1.4, 1.6 kA Current duration: 10 s	Grade 1 Ti alloy sheets Length: 120 mm Width: 10 mm Thickness: 1 mm	Li et al. [74]
Preload 100 N Compression rate: 24 mm/min Max displacement: 9 mm Compression force: 41 kN	Continuous and pulsed DC Current density: 20, 28 A/mm^2^ Current duration: 5, 1.1 s	SUS316L and IN718 bars, *ϕ*10 × 15 mm	Li et al. [75]
Preload 100 N Compression rate: 23.5 mm/min Max displacement: 9 mm Compression force: 41 kN	Continuous and pulsed DC Current: 2.2, 1.6 kA Current duration: 5, 1.1 s	SUS316L and IN718 bars, *ϕ*10 × 15 mm	Anaman et al. [40]
Compression rate: 24 mm/min Max displacement: 1.2 mm Compression force: 8–12 kN	Continuous and pulsed DC Current density: 60,65,75,80 A/mm^2^ Current duration: 3, 7 s	S45C and AL6061-T6 sheets Thicknesses: 2 mm Width: 10 mm Length: 100 mm	Zhang et al. [42]
Preset compressive loads: 20 kN (Case I) 40 kN (Case II Two-stage experiments: Stage I- Joining with forming Stage II- Forming with tempering Cooling time between Stage I and II: 20 s	Pulsed DC **Case I:** Stage I pulses: 8.0 kA–0.35 s followed by 6.5 kA –1 s (four times) with an interval of 0.2 s Stage II pulse: 8.5 kA–1.0 s (four times), **Case II:** Stage I pulses: 9.5 kA–0.35 s followed by 6.5 kA –1 s (four times) with an interval of 0.2 s Stage II pulse: 8.5 kA–1.2 s (four times)	Q235B steel ball SCM435 steel stud	Do et al. [76]
Compressive displacement: 0.167 mm/s	Continuous DC Current density: 36 A/mm^2^	Functionally graded austenitic Fe–Mn–Al–C lightweight steels doped with Mo and Si, respectively Cylindrical specimens Diameter: 9 mm Height: 9 mm	Lee et al. [43]

The EA pressure joining of two dissimilar, identical solid cylindrical specimens of stainless steel SS316L (SUS316L) and Inconel 718 (IN718) was experimentally investigated by Li et al. [75]. With the application of continuous DC of density 28 A/mm^2^ for 5 s, followed by pulsed DC of density 20.37 A/mm^2^ for 1.1 s in a period of 35 s, they fabricated void-free joints without melting and solidification revealed from microstructural analysis. The defect-free joints between SUS316L and IN718 were achieved with diffusion zone thicknesses of approximately 1.2 μm for a 0.5-s holding time (H-0s) and 1.9 μm for a holding time of 36.5 s (H-36s), indicating enhanced atomic diffusion with longer current duration. For the H-0s joint, the IN718 grain size was reduced to 7.8 ± 7.1 μm, which is approximately a 76% reduction compared to the base metal grain size (32.8 ± 13.2 μm). For the H-36s joint, the IN718 grain size was further reduced to 2.8 ± 1.5 μm, reflecting about a 91% reduction from the base metal grain size. This grain refinement led to increased hardness on the IN718 side, with peak values reaching ~ 450 HV, higher than that of the base metal. Under tensile testing, fractures consistently occurred in the SUS316L side with large plastic deformation and ductile fracture characteristics. A subsequent work by Anaman et al. [40] also considered the EA pressure joining of the same dissimilar cylindrical specimen using the same current to characterize the electrochemical properties of the joint. The study showed that the joint behaves in a galvanic manner: the SUS316L side corroded when the joint was exposed to a 3.5% NaCl electrolyte solution at room temperature, leaving the IN718 intact. The corrosion resistance of the joint was decreased due to the microstructural changes that occurred during the joining process.

Zhang et al. [42] studied the feasibility of EA pressure lap joining of dissimilar steel S45C and aluminum 6061-T6 alloy employing both continuous and pulsed DC densities of magnitudes varying from 60 to 80 A/mm^2^. They observed that the joint strength increases with increasing current density and holding time at the elevated temperature. Further, they report that the fracture load increased with increasing electric current density up to 75 A/mm^2^; thereafter, the fracture load slightly decreased. The application of EA pressure joining to the simultaneous joining and forming of dissimilar steels Q235B (ball) and SCM435 (stud) was experimentally demonstrated by Do et al. [76] by applying compressive deformation and pulsed electric current simultaneously. They report the maximum bending load to break the fabricated ball studs to be higher than the target value requested for commercialization. It was reported that the EA pressure joining for the said application leads to grain refinement, induces tempered martensite, and increases the portion of polygonal ferrite in the deformed zone, the joining zone, and the thermal-mechanically affected zone (TMAZ), respectively. Vickers microhardness measurements showed significant variation across regions, with values of 859.3 ± 5.8 HV at the ball surface, 341.2 ± 4.2 HV at the stud base metal, and a minimum in the TMAZ.

Recently, a functionally graded austenitic lightweight steel (LWS) was fabricated through EA pressure joining of Mo-doped and Si-doped lightweight steels by Lee et al. [43] at a peak temperature of 1122 °C, well below their melting points (1283 °C for Mo-LWS and 1247 °C for Si-LWS), confirming a solid-state bond. The microstructural characterization revealed the presence of a unique κ-carbide gradient structure with the formation of a homogeneous metal matrix at the joint. Investigating the joint properties via joint strength tests, the EA pressure jointed part was reported to exhibit superior mechanical properties, such as a yield strength of 500 MPa, ultimate tensile strength of 770 MPa, and 23% total elongation, with the Mo-doped region alone elongating up to 40%, indicating both high strength and localized ductility.

Although the preceding literature investigates the EA pressure joining of two dissimilar materials, nevertheless, a few experimental studies have considered the EA pressure joining of similar materials. The EA pressure joining of 316L stainless steel cylindrical specimens using an additively manufactured metal porous interlayer between joining specimens was experimentally demonstrated by Hong et al. [73]. During the experiment, a continuous DC of density 32 A/mm^2^ for a duration of 5 s, followed by a pulsed DC of density 19 A/mm^2^ for a duration of 0.75 s over a pulse period of 1.25 s, was applied directly to the joint under continuous compression in the axial direction. A defect-free joint was obtained using a lower compressive force. The microstructure analysis revealed the absence of material porosity at the joint cross-section due to compressive deformation and recrystallization during joining. Further, the authors reported that the mechanical properties, such as microhardness and bending strength, of the joints with interlayers are comparable to those without interlayers. A successful sound solid-state joint at a temperature significantly lower than the melting temperature was obtained by Li et al. [74] in EA pressure joining between grade 1 titanium alloy sheets by applying continuous DC of very high magnitudes, ranging from 1.2 to 1.6 kA for a duration of 10 s. Based on the shear tensile test, they concluded that the optimal thickness reduction corresponding to the maximum fracture load decreases as current intensity increases.

#### EA friction stir welding

**Fig. 3:**
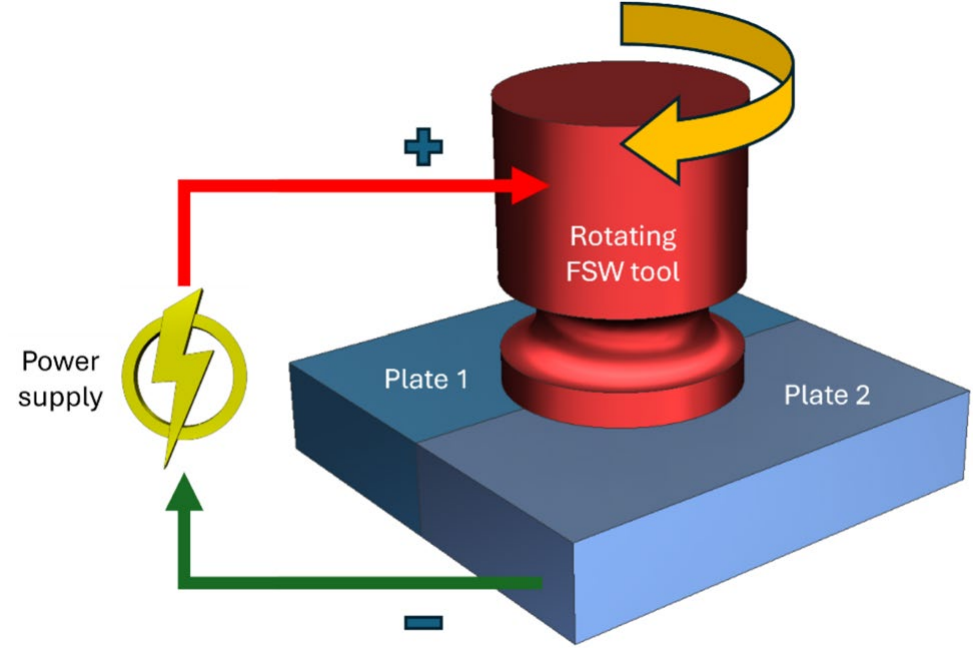
Schematic of EA-FSW process

EA friction stir welding (EA-FSW) was developed as one of the new hybrid joining processes to increase the efficacy of conventional friction stir welding (FSW) of high-melting temperatures of similar or dissimilar materials for different industrial applications. In this process, electric current is applied to the joining interface between the two materials through the rotating FSW tool that is inserted into the interface and moves forward along the joint line as depicted in Fig. 3. The process combines the beneficial effect of electroplasticity and Joule heating due to flow of electric current with the frictional heat generated by the rotating tool to increase softening and plasticizing of the materials at the joint interface. This amounts to reducing the welding force in building a sound, solid-state joint. Research works attempting to investigate the EA-FSW of various similar and dissimilar materials experimentally are summarized in Table 4.

**Table 4:** EA friction stir welding

Process parameters	Current	Specimen	Reference
Welding speed: 60 mm/min	Continuous DC Current: 560 A	TRIP 780/800 and Al6061-T6511 sheets Thickness: 1.4 mm	Liu et al. [77]
Rotating speed: 800 rpm Welding speed: 160 mm/min	Continuous DC Current: 100, 200, 300, 400, 500, 600 A	Al AA2219 T87 Dimensions: 180 × 100 × 6 mm	Chen et al. [78]
Rotating speed: 1300 rpm Welding speed: 50 mm/min	Continuous DC Current: 100, 200 A	AZ31B Mg Dimensions: 22 × 10 × 5 mm	Han et al. [79]
Rotating speed: 200 rpm Welding speed: 40 mm/min	Continuous DC Current: 100, 200 A	Ti-6Al-4V Thickness: 3 mm	Jiang et al. [41]

A novel EA-FSW system was developed by Liu et al. [77] using two separate copper brush electrodes to introduce electric current to the FSW process without involving the tool in the electric circuit. The system was used to investigate the effectiveness of the electroplastic effect in FSW of dissimilar Al 6061 to TRIP 780 steel by supplying a continuous DC of 560 A using a Lincoln Electric Power Wave 455 power source. The authors reported that both the electroplastic effect and Joule heating reduce axial welding force. The electroplastic effect was observed to be more significant at lower rotating speeds and smaller tool offsets. They also performed microstructural analysis of the EA friction stir welded joint and observed improved joint quality with the formation of micro interlock features in welding sections, which help restrain crack initiation in the brittle intermetallic compounds layer.

Chen et al. [78] carried out an experimental study of EA friction stir joining of 2219 aluminum alloy plates applying continuous DC ranging from 0 to 600 A. An experimental system where the current was applied to the joint interface via the FSW tool was used. The authors observed that the introduction of current increased the overall hardness of the weld zone as compared to the conventional friction stir welded joint. They reported an improvement of 2.74–7.38% in the tensile strength of the joint for currents 100–400 A, albeit this improvement was significantly higher, of the order of 17.11% for currents 500 A and 600 A. Further, they observed the change in fracture location of the joint from the interface of the nugget zone/thermo-mechanically affected zone at the advancing side in FSW, to the nugget zone at the retreating side in EA-FSW, leading to V-shaped ductile fracture. A similar experimental arrangement was also used by Han et al. [79] and Jiang et al. [41] to study the EA-FSW of AZ31B magnesium alloy plates and Ti-6Al-4V titanium alloy plates, respectively, supplying continuous DC varying from 0 to 200 A. Han et al. [79] reported that increasing current from 0 to 200 A enhances joint quality by enlarging and reshaping the stir zone (SZ) and reducing grain size variation across thickness from 6.44–6.63 to 4.32 µm. Tensile strength improved by up to 7.2%, reaching 210.6 MPa, which is 84.95% of base material strength, while fracture locations shifted from the SZ/TMAZ boundary to the center of SZ due to improved strength uniformity. However, the hardness of the joint was not affected by the current. On the other hand, Jiang et al. [41] reported that larger grain size decreases hardness in the stir zone, while smaller grain size and decreasing dislocation density increase hardness in the heat-affected zone in the FSW joint of Ti-6Al-4V alloy from 200 to 100 and 0 A. For 100 A current, the joints contained smaller macrozones, leading to better fatigue performance than conventional FSW.

### EA metal forming processes

To realize efficient forming of various difficult-to-form materials, e.g., aluminum, titanium, and magnesium alloys in different industrial applications such as lightweight automotive and aerospace vehicles, the use of an electric field was proven as a promising technique. This innovative way of forming materials with the aid of electric current is known as EA metal forming or EA forming processes. The EA forming processes have shown significant improvements over the conventional forming processes in terms of reducing deformation forces, enhancing formability by reducing flow stress and eliminating the springback effect. In this section, the current state-of-the-art on various EA forming processes is presented.

#### EA rolling

**Fig. 4:**
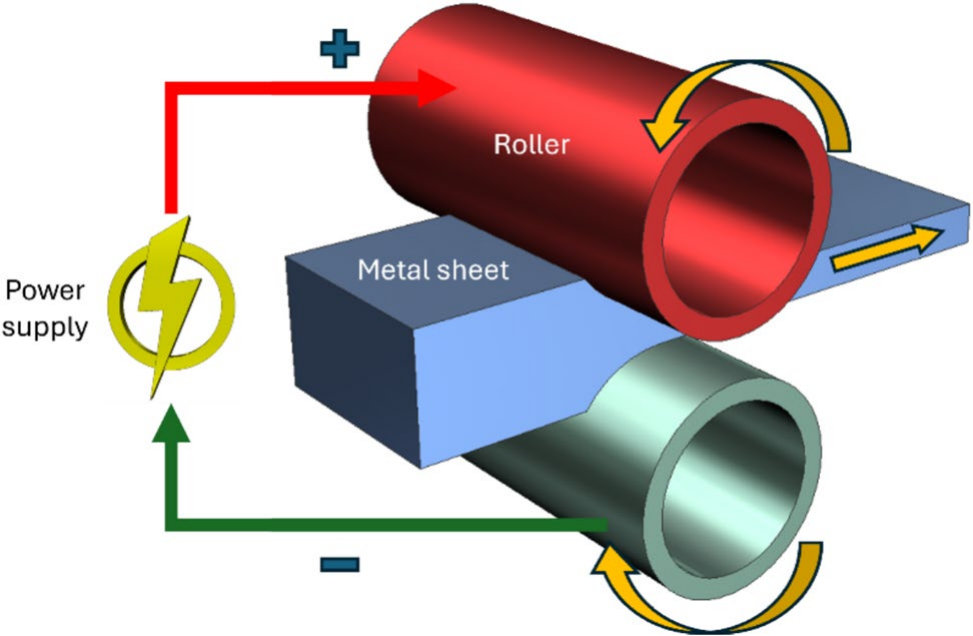
Schematic of EA rolling

The principle of the EA rolling process is schematically illustrated in Fig. 4, comprising two counter-rotating rollers made of electrodes while a metallic strip is being passed through them to deform electroplastically. The use of pulsed electric current for the ageing and cold rolling of magnesium AZ91 alloy strips was proposed by Jiang et al. [80] to improve mechanical properties. In their experimental setup, the strip was passed between two roller electrodes employing a DC pulsed current of density in the range 327–336 A/mm^2^ with a pulse width of 70 μs. They report that the ultimate tensile strength, yield strength and elongation to failure of the EA rolled samples were enhanced by 11–12%, 10%, and 70–75%, respectively, when compared to the original extruded strip.

The rollability of magnesium AZ31 alloy sheet in EA rolling was experimentally studied by Kuang et al. [81, 82]. In [81], the effect of electric current in a single pass large strain EA rolling of the sheet was investigated using a pulsed electric current of different densities in the range 0–17.9 A/mm^2^. The critical current density of 9.8 A/mm^2^ was reported to achieve transverse-direction split texture, enhancing further formability of the alloy. A higher current density resulted in a more significant transverse-direction split texture. In [82], the comparative rollability of the alloy in a multi-pass EA rolling and conventional warm rolling using pulsed electric current densities varying from 16.4 to 21.5 A/mm^2^ was studied. The use of pulsed electric current in EA rolling showed improved rollability compared to warm rolling. The EA rolling of the sheet succeeded with no edge cracks up to an accumulated true strain of −1.09. However, the warm rolled sheets cracked at an accumulated true strain of −0.24. The authors attributed this rollability enhancement in EA rolling to the improved homogeneity of plastic deformation and dynamic recrystallization, which postponed the crack initiation. Further, they argued that at high accumulative strains, the refined grains and modified texture prevent stress concentrations.

In a study by Kuang et al. [83], the EA rolling of AZ31 alloy against conventional warm rolling at the same deformation temperature was compared, and this revealed an athermal electroplastic influence of the pulsed electric current on different twinning behavior, improving the rollability of the sheet. They reported that cracks in conventional warm rolled samples appeared after the second rolling pass at an accumulative true strain of −0.25, while EA-rolled samples remained crack-free and free of macroshear bands even after the sixth pass at an accumulative true strain of −1.05.

Li et al. [84] achieved a heterogeneous structure in hard-to-deform hcp Zr through EA rolling using DC pulsed current densities in the range of 80–100 A/mm^2^, followed by low-temperature annealing. It was observed that the electropulsed Zr exhibited a significantly refined microstructure with ultrafine grains after annealing, which resulted in superior mechanical properties. After annealing at 450 °C for 1 h, the electropulsed Zr reached an ultimate tensile strength of ~ 617 MPa and a uniform elongation of 12.3%, whereas the non-electropulsed Zr exhibited a reduced strength of ~ 460 MPa and lower ductility. Similar observations were made by Xiao et al. [33] in the case of Al–Mg-Li alloy sheets produced through cold rolling combined with electropulsing-assisted recrystallization annealing and electropulsing-assisted ageing using an in-house developed setup. They reported enhancements in the yield strength, ultimate tensile strength, and elongation to fracture of the electropulse-treated sheets as 29.1 ~ 35.1%, 15.7 ~ 21.6%, and 23.3 ~ 68.5%, respectively, compared to the alloy treated by conventional heat treatment techniques. The enhancements in mechanical properties were mainly due to electropulsing-promoted recrystallization, resulting in the rapid formation of uniformly distributed fine grains. The grain size was refined from Hundreds of micrometers to about 10~26 μm.

Guo et al. [85] introduced a constant DC pulse current in the EA rolling of TC4/SS304 plates to investigate the interface bonding strength of the composite plate. Rolling was performed at 750 °C with and without current, using a 19.5% reduction in the first pass and cumulative reductions of 38% and 58% in subsequent passes. In the absence of current, conventional rolling failed to establish metallurgical bonding with interface strength attributed primarily to mechanical interlocking. On the other Hand, the application of pulsed current enabled full metallurgical bonding even at the initial 19.5% reduction, attributed to enhanced atomic diffusion and localized interface heating. The study further examined the effect of temperature on EA rolling at a fixed 58% reduction, revealing the maximum strength of 239 MPa at 650 °C, which decreased to 177 MPa at 750 °C and dropped sharply to 92 MPa at 850 °C due to microvoid formation and brittle intermetallic compounds. The highest overall bonding strength of 286 MPa was observed at 38% reduction and 750 °C.

Recently, using an electropulsing power supply system (CTNP 1621–50/3000FN) and a double working roller mill, the EA rolling of electropulse-bonded Al/Cu laminated metal composites was carried out by Ran et al. [86]. They report that electropulsing accelerates the diffusion of Al and Cu atoms significantly, facilitating the formation of the diffusion layer. The thickness of the diffusion layer increases with the increase of the electropulsing frequency. It was found that the maximum diffusion coefficient of the electropulsing-treated sample is approximately 6.5 times higher than the conventional hot-rolled sample under similar processing temperatures. Through EA rolling, the maximum electroplastic effect was achieved at a frequency of 20 kHz, yielding the maximum tensile strength, ductility and significantly enhanced recrystallization behavior of the Al and Cu layers with refined grain size. The maximum tensile strength obtained was ~ 215.2 MPa, and the maximum elongation achieved was ~ 52.4% for the EA rolled samples at 20-kHz frequency. The summary of the process parameters used in the EA rolling of various materials is presented in Table 5.

**Table 5:** EA rolling

Process parameters	Current	Sample	Reference
Rolling speed: 2 m/min Distance between electrodes: 225 mm	Pulsed DC, Current density: 327–336 A/mm^2^ Pulse width: 70 μs Frequency: 95–220 Hz	Magnesium alloy AZ91 sheet Width: 2.90 mm Thickness: 1.45 mm	Jiang et al. [80]
Roller’s diameter: 60 mm, Distance between electrodes: 225 mm Rolling speed: 1.5 m/min	Pulsed DC Current density: 16.4–21.5 A/mm^2^ Pulse width: 60 μs Frequency: 250–400 Hz	Magnesium alloy AZ31 sheet Length: 1000 mm Width: 11 mm Thickness: 1.6 mm	Kuang et al. [81]
Roller’s diameter: 60 mm Distance between electrodes: 200 mm Rolling speed: 0.8 m/min	Pulsed DC Current density: 0–17.9 A/mm^2^ Pulse width: 60 μs Frequency: 300 Hz	Magnesium alloy AZ31 sheet Thickness: 1.6 mm	Kuang et al. [82]
Roller’s diameter: 60 mm Distance between electrodes: 200 mm	Pulsed DC Current density: 17.9 A/mm^2^ Pulse width: 60 μs Frequency: 400 Hz	Magnesium alloy AZ31 sheet Thickness: 1.6 mm	Kuang et al. [83]
Strain: 2.83 Strain rate: 1.6 s^−1^	Pulsed DC Current density: 80–100 A/mm^2^ Voltage: 27 V Frequency: 500 Hz Pulse width: 80 μs	Pure Zr Sheet Thickness: 2.7 mm	Li et al. [84]
Reduction ratio: 66.7%	Pulsed DC Current density: 9.6–21 A/mm^2^ Frequency: 100 Hz Pulse width: 5 ms	Al–Mg-Li alloy sheet Length: 70 mm Width: 10 mm Thickness: 1.5 mm	Xiao et al. [33]
Reduction ratios: 19.5%, 38% and 58% Rolling temperatures: 750 °C (at 19.5% and 38% reduction ratios) 650 °C, 750 °C, 850 °C (at a 58% reduction ratio)	Pulsed DC square wave Current: 350 A Frequency: 500 Hz Duty cycle: 50%	SS304/TC4 plates SS304: 60 mm × 150 mm × 3.5 mm TC4: 60 mm × 150 mm × 2 mm	Guo et al [85]
Reduction ratio: 10%	Pulsed DC Current intensity: 500 A Frequency: 10, 20, 30 kHz Duty cycle: 50%	Electropulse-bonded Al/Cu laminated metal composites Length: 50 mm Width: 50 mm Thickness: 1 mm	Ran et al [86]

#### EA forging

**Fig. 5:**
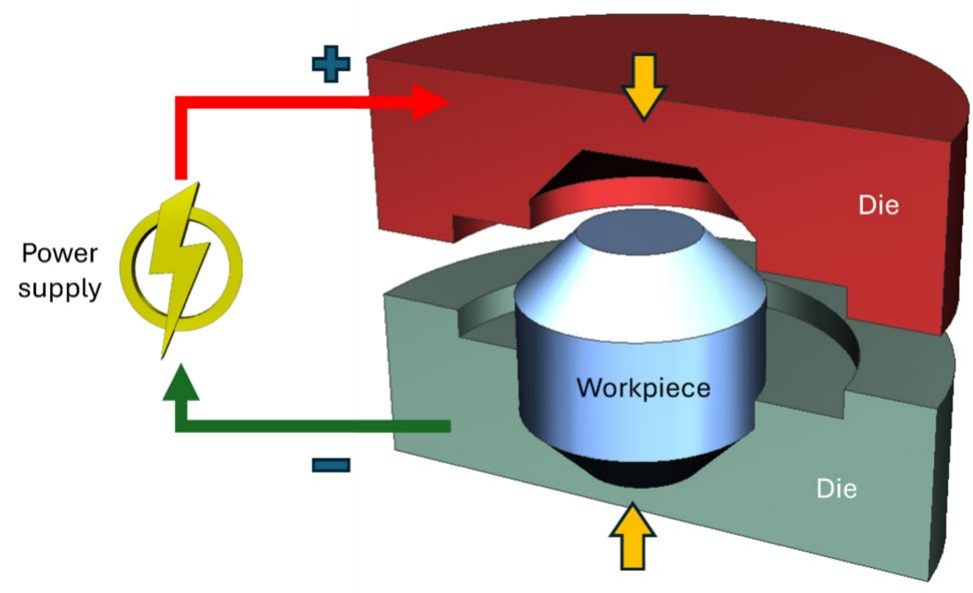
The principle of EA forging

In EA forging, electric current is passed to the specimen concerned through two compressive dies as shown in Fig. 5. The feasibility of EA-progressive forging, as well as EA-continuous forging of aluminum 6061-T6 alloy, was experimentally studied by Hong et al. [87], by applying DC pulsed electric current produced by a Vadal SP-1000U welder generator. For the progressive forging, electric current densities of 75 and 90 A/mm^2^ with a short duration of 0.5 s were applied to the specimen after each progressive compressive displacement. For the EA-continuous forging, the above current parameters were applied during continuous deformation of the specimen over a pulse period of 29.5 μs. The authors observed improved formability in both EA-progressive and EA-continuous forging with reduced average compressive load for the alloy. The more prominent improvement in formability of the alloy was achieved in EA-progressive forging with reduced compressive load, albeit the temperature of the specimen was lower than that of EA-continuous forging during deformation. Based on the microstructural analysis, they conclude that electrically induced annealing occurred in EA forging due to the application of electric current. The experimental results confirmed the effect of electroplasticity in improving formability in EA forging, which is distinct from Joule heating.

The EA forging of bearing steels, such as M50, M50 Nil, and Pyrowear P675 was experimentally studied by Lang et al. [88] using DC continuous current of different densities generated by a Lincoln R35 Arc Welder. Their study demonstrated a clear reduction in compressive load with the application of electric current. Compression tests revealed threshold current densities of 33 A/mm^2^ for Pyrowear P675, 48 A/mm^2^ for M50, and 37 A/mm^2^ for M50 NiL, resulting in strain reductions of approximately 25% for Pyrowear P675 and 50% for both M50 and M50 NiL compared to no-current conditions. In addition, the application of current increased both springback and hardness. In particular, M50 exhibited Rockwell hardness gains of up to 18 HRC at 48 A/mm^2^, suggesting that phase transformations may have occurred during deformation. The process parameters associated with the above studies are summarized in Table 6.

**Table 6:** EA forging

Process parameters	Current	Specimen	Reference
Uniaxial compression Displacement rate: 2 mm/min	Pulsed DC Current density: 75, 90 A/mm^2^ Pulse width: 0.5 s Frequency: 0.0338 Hz	Aluminum 6061-T6 alloy Cylindrical specimen Diameter: 10.2 mm Height: 15 mm	Hong et al. [87]
Uniaxial compression Displacement rate: 16.5 mm/min Strain rate: 1.75 min^–1^	Continuous DC Current densities: 0, 15, 26, 36, 48 A/mm^2^ (for M50) 0, 15, 25, 34, 38 A/mm^2^ (for M50 Nil) 0, 15, 23, 33, 37 A/mm^2^ (for Pyrowear P675)	M50, M50 Nil, and Pyrowear P675 Cylindrical specimen Diameter: 6.35 ± 0.025 mm Height: 9.525 ± 0.127 mm	Lang et al. [88]

#### EA drawing

In EA drawing, electric current is imposed in the component in a conventional drawing system. Here, we explore recent studies in electrically-assisted conventional wire drawing (a bulk deformation process) as well as deep drawing (a sheet metal forming process).

##### EA wire drawing

**Fig. 6:**
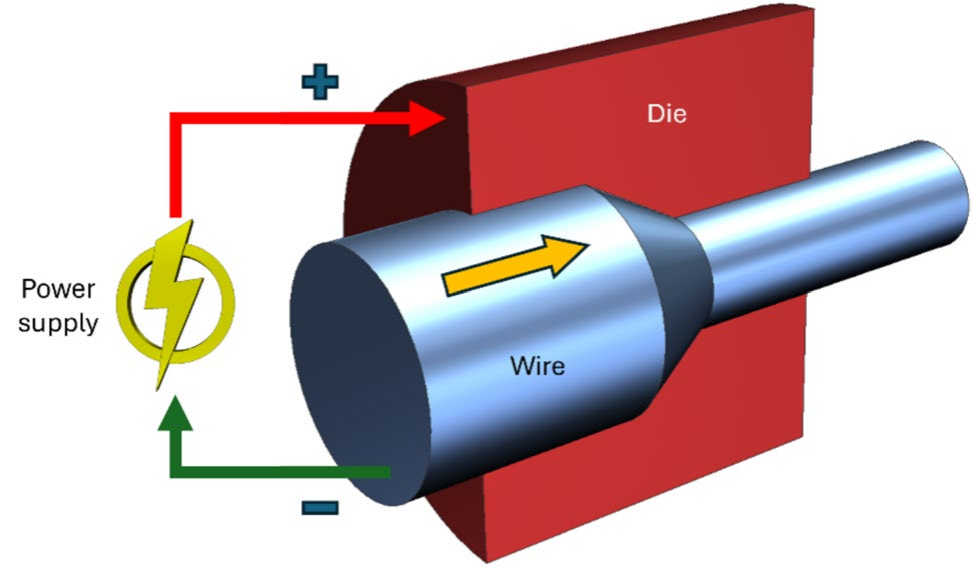
Schematic of EA wire drawing process

In EA wire drawing, electropulsing is introduced to the wire workpiece, supplying current via a drawing die as shown in Fig. 6, while the wire is subjected to severe plastic deformation due to the application of a constant tensile force to form into the desired reduced cross-section. Usually, in the design of an electric circuit in EA wire drawing, the wire is made as the positive electrode (anode) and the drawing die is made as the negative electrode (cathode). The different investigations on the EA wire drawing process are summarized in Table 7. In general, the investigations primarily focused on the effect of electric current on drawing force, elongation, and microstructural modifications.

**Table 7:** EA wire drawing

Process	Current	Specimen	Reference
Diamond dies Angle:* α* = 9° Diameter: 60–113 μm Drawing speed: 5–200 m/min	Continuous and pulsed DC Current density: 250–3000 A/mm^2^ Pulse width: 150–200 μs Frequency: 600–800 Hz	Mob-60 copper alloy 12X18H10T steel alloy	Troitskiy and Stashenko [89]
Conic dies Angle: *α* = 6° Diameter: 0.753 mm Drawing speed: 0.5 m/min	Pulsed DC Current intensity: 312 A Current density: 184.6 A/mm^2^ Pulse width: 100, 150, 200, 250 μs Frequency: 142, 177, 237, 355 Hz Average power: 800 W	308L stainless steel Length: 700 mm Diameter: 175 mm	Egea et al. [90]
Single pass drawing Reduction of wire diameter: From 0.8 mm to 0.7 mm Drawing speed: 10 mm/s Lubricant used	Pulsed DC Pulse width: 40 μs Current densities: 1000, 2100, 2600, 3000 A/mm^2^	Pure Cu Prior to test, annealed for 30 Min at 700 K Grain size: 25 μm	Dalong et al. [91]
Conic dies Angle: *α* = 6° Drawing speed: 0.5 m/min	Pulsed DC Current density (RMS): 34.8 A/mm^2^ Pulse width: 250 μs Frequency: 142 Hz	308L stainless steel Diameter: 1.60 mm	Egea et al. [34]

Troitskiy and Stashenko [89] studied the feasibility of EA wire drawing of copper and steel wires and compared them with the corresponding conventional and warm wire drawing. They conducted EA wire drawing experiments by applying both DC continuous and pulsed electric currents and reported that the use of DC pulsed current was better than continuous current in terms of reducing the drawing force. The study suggests that the polarity of current matters. The reversing of polarity can have low to no effect. A reduction of 30–35% in the drawing force and of 18–20% in electrical resistance, an increase in plasticity, a decrease in breakage rate and tensile strength of the wires were observed due to the electroplasticity effect induced by pulsed electric current in EA wire drawing compared to the warm drawing assisted by alternating current. The microstructural study suggested that possible heating from electropulsing led to dynamic recovery and dynamic recrystallization. The effect of electroplasticity was not observed in warm drawing with alternating current.

Inspired by the work of Troitskiy and Stashenko [89], the electroplastic drawing force in EA wire drawing of pure copper under pulsed electric current was experimentally assessed by Dalong et al. [91]. Apart from experimental appraisal, they also developed an analytical model to predict the electroplastic drawing force. They reported a reduction in drawing force aided by the effect of electroplasticity along with Joule heating, the vibration impact of the pulsed current, the influence of electric current on the surface work function, and the self-generated thermoelectric potential. The reduction in drawing force was also observed by Egea et al. [90] in a numerical and experimental study of EA wire drawing of 308L stainless steel under different electropulse configurations. The study found that longer pulse durations and lower frequencies reduce drawing forces more effectively, with a maximum reduction of 10.6%, from 582.8 N in the conventional process to 520.8 N using a 142 Hz/250 μs pulse setting. In addition, the EA drawing reduced von Mises stress from 1200 to 1057 MPa at the wire surface, decreased yield strength by 150 MPa, and increased elongation by approximately 10%, indicating enhanced formability compared to conventional drawing. However, this achievement was reported to be independent of the pulse duration and frequency configuration used. Further, the authors reported that electropulses in EA drawing decrease hardness, causing a dynamic recrystallization, a detwinning, an attenuation of *α* martensite, and an increase in grain size. In a later study by Egea et al. [34], similar observations were reported on the alteration of microstructures of commercial 308L stainless steel induced by electropulses in EA wire drawing while comparing microstructures obtained in conventional thermal annealing treatment.

##### EA deep drawing

In the last decade, a number of studies were devoted to investigating the EA drawing of different alloy sheets of industrial importance using either electric current or electric current in conjunction with a magnetic force. These investigations are summarized in Table 8.

**Table 8:** EA deep drawing

Process	Current	Specimen	Reference
Electrically assisted cylindrical deep drawing	Pulsed DC Current: 60 A Pulse width: 60 μs	Mg AZ31B sheet Thickness: 1.5 mm	Xie et al. [92]
Electropulse-assisted deep drawing	Pulse DC Effective current: up to 50 A	Low carbon steel Diameter: 78 mm	Lv et al. [93]
Incremental electromagnetic-assisted stamping (IEMAS) with radial magnetic pressure supported by FEM simulations	Up to 53.4 kA	Al alloy 5052-O sheets Diameter: 200 mm Thickness: 1 mm	Cui et al. [59]
Electromagnetic pulse-assisted incremental drawing (EMPAID). Experiments and FE simulations	Electromagnetic pulse generator Capacitance: 213 μF Discharge voltage: up to 30 kV Resistance: 3.35 mΩ Inductance: 12.24 μH	Al AA5052-O sheet Diameter: 200 mm Thickness: 1 mm	Fang et al. [94]
Radial Lorentz force augmented deep drawing using a dual-coil electromagnetic forming (EMF) system	Effective force pulse width: 220–286 μs	Al 1060-H24 sheet Diameter: 130 mm Thickness: 1.35 mm	Lai et al. [95]
Radial Lorentz force augmented deep drawing experiments and FE simulations (LS-DYNA)	Discharge voltage for coil 1 (radial Lorentz force driving coil): 3 kV, 4.5 kV, and 6 kV Discharge voltage for coil 2 (axial Lorentz force driving coil): 0 kV, 2 kV, 4 kV, 6 kV, and 8 kV	AA1060-O Al alloy sheet Diameter: 130 mm Thickness: 1.5 mm	Chen et al. [60]

An EA cylindrical deep drawing of AZ31B magnesium alloy sheet to assess the effect of pulsed electric current on its ductility was studied experimentally by Xie et al. [92]. They used a customized DC electric pulsed generator to apply a current of 60 A with a pulse width of 60 μs through the flanges and die corners. The results demonstrated a significant increase in the deep drawing limit, from 3 mm at 298 K to 11.5 mm at 448 K. Additionally, a 1 mm increase in drawing depth was achieved by increasing electric pulse frequency or peak current at a constant temperature, confirming the electroplastic effect. Microstructural analysis revealed that the electric pulses induced dynamic recrystallization at 398 K, improving ductility through accelerated nucleation.

Further, Lv et al. [93] carried out experiments on EA deep drawing of high-strength low-carbon steel by using a square waveform DC pulse power supply with an effective current of up to 50 A and introducing pulse electrodes into the drawing die. The application of a 46 A pulse current for 300 s increased the deep drawing limit of 650-MPa steel from 13 to 14 mm, without any crack formation. The process also improved the surface quality of 920-MPa steel by eliminating cracks at 8.8 mm, compared to 8.3 mm in conventional drawing. A maximum temperature difference of approximately 26 °C was observed across the sheet, with the center reaching the highest temperature due to Joule heating. Hardness increased significantly at the flange area, reaching up to 351 HV, with gains of 47.5% and 34% for the 650-MPa and 920-MPa steels, respectively, while changes at the cylinder bottom remained minimal. Microstructural analysis revealed that the ferrite-to-martensite ratio remained stable; however, ferrite grains elongated considerably, as indicated by an increase in length-to-width ratio from ~ 1 before drawing to 2 (650 MPa) and 3.8 (920 MPa) in the straight wall region, correlating with localized hardness improvements.

The use of electromagnetic fields in the deep drawing of aluminum alloy sheets was proposed as a new EA deep drawing method. An experimental investigation of an innovative EA deep drawing, viz., incremental electromagnetic-assisted stamping with radial magnetic pressure for cup drawing of aluminum 5052-O alloy sheet supported with three-dimensional (3D) finite element method (FEM) simulations was carried out by Cui et al. [59]. The electric current flowing through the coil during experimentation was up to 53.4 kA. The authors reported an increase in the forming depth compared to traditional stamping of 13% with one coil discharge and 31% with three coil discharges. The FEM simulations revealed the generation of a radial magnetic force at the sheet center when placing the coil at the end of the sheet. This causes a material flow from the sheet flange to the region corresponding to the corner of the die after the coil discharge, ensuring the formation of deeper cylindrical cups, without becoming too thin in certain regions. The electromagnetic-assisted deep drawing of the same aluminum alloy sheet was also experimentally studied by Fang et al. [94], supported by FEM simulations. They proposed the use of alternating electromagnetic pulse forces generated by an electromagnetic pulse generator. They reported that the limit drawing height at a constant sheet/punch diameter ratio was increased by 116% using the proposed method as compared to conventional drawing.

Radial Lorentz force augmented deep drawing of aluminum 1060-H24 sheet with an effective force pulse width of 220–286 μs, using a dual coil electromagnetic forming system, was proposed by Lai et al. [95]. The authors have developed an electromagnetic and structure-coupled numerical model using MATLAB and ANSYS to simulate the proposed process. They observed that increasing the discharge voltage of the axial Lorentz force driving coil linearly increased the draw-in and forming height; these features increase exponentially if the discharge voltage of the radial Lorentz force driving coil is increased. The same process was also studied experimentally and numerically for an aluminum AA1060-O alloy sheet by Chen et al. [60], who reported that the forming shape of the deformed workpiece can be altered from convex to flat or concave by increasing the discharge voltage of the radial Lorentz force driving coil. Increasing the discharge voltage of the axial Lorentz force driving coil enhances the draw-in.

#### EA stretch forming

To manufacture large-size and thin-walled ellipsoidal parts, an electromagnetic incremental forming process combined with stretch forming was proposed by Cui et al. [96]. They used two small working coils moving along a 3D trajectory, discharged at different positions with a capacitance of 213 μF and a rated voltage of 30 kV to deform the sheet locally. They also carried out a 3D FEM analysis of the proposed process to assess the effect of the number and moving path of coils on the formability of aluminum AA 3003 sheet, supported with experimental validation. The sheet was first stretched by moving down the pressing plate, and then coils discharged to make contact with the die. Significant improvements were noted in the uniformity of the sheet deformation when the symmetrically arranged double coils were discharged in two circles for each layer. Further, they achieved a more uniform thickness distribution in the sheet formed by the proposed method as compared to conventional incremental forming. The maximum tensile radial and hoop stresses occurred in the sheet at a time of 200 μs and then released and shocked after 370 μs, causing a small gap between the sheet and the die after the coil discharge.

In a recent study, Cui et al. [36] carried out a 3D FEM simulation using ANSYS and an experimental study of an electromagnetic stretch forming process to minimize the springback while forming a large thin-walled single curvature of AA3003-H14 alloy sheet. In the experiment, they used a 200-kJ electromagnetic forming machine with two large working moving coils. Pulsed discharge current was passed through the coils at a maximum discharge voltage of 25 kV and maximum capacitance of 640 μF. They observed little springback occurring in the formed sheet after several discharges, as compared to springback in simple bending with a large bending radius. They also reported the generation of small residual stresses in the electromagnetically stretched formed sheet. A summary of the process parameters used in the above studies is provided in Table 9.

**Table 9:** EA stretch forming

Process parameters	Current	Specimen	Reference
Pressing plate and the support Plate: hole radius 390 mm and corner radius 10 mm Diameter of the sheet: 1150 mm Symmetrically arranged two flat spiral coils with the following dimensions: Wire cross-sectional area: 3 mm × 6 mm Inner coil radius: 341 mm Separation distance: 4 mm	Discharge from capacitor banks Voltage: 7 kV Peak current: 25 kA Capacitance: 213 μF Resistance: 50 mΩ Inductance: 11.73 μH	Aluminum AA 3003 alloy sheet Thickness: 1 mm	Cui et al. [96]
Clamping force: 40 kN Rectangular spiral coil consisting of 2 × 5 mm^2^ copper wires	200 kJ EM machine Discharge voltage: 25 kV Capacitance: 640 μF	AA3003-H14 alloy sheet Length: 750 mm Width: 150 mm Thickness: 1 mm	Cui et al. [36]

#### EA incremental forming

The EA incremental forming has proven to be an efficient and cost-effective technique for the forming of difficult-to-form metallic sheets over the conventional incremental forming process. Table 10 highlights the recent research works on EA incremental forming.

**Table 10:** EA incremental forming

Process	Current	Specimen	Reference
Toor diameter: 8 mm Step size: 0.3 mm Feed rate: 1000 mm/min Form: truncated cone Diameter: 60 mm Wall angles: 45°, 56°, 64°	Continuous DC Current intensity: 400 A	Ti -6Al-4V sheets Size: 90 mm × 90 mm × 1 mm	Honarpisheh et al. [97]
Tool radius: 6 mm Feed rate: 750−1500 mm/min Step size: 0.5 mm Form: squared pyramids Heights: 19 − 23 mm Wall angles: 40°, 25° Base: 90 × 90, 75 × 75 mm	Continuous DC Current intensity: 437.9–519.8 A	CP-K 60/78, DP1000 steel sheets Thickness: 0.8–1 mm Size: 280 mm × 280 mm	Min et al. [98]
Four tools comparison Tool radius: 5 mm Step size: 0.3 mm Feed rate: 800 mm/min Form: truncated cone Diameter: 90 mm Forming depth: 20 mm Wall angle: 45	Continuous DC Current intensity: 200–300 A	Ti-6Al-4V sheets Size: 180 mm × 180 mm × 1 mm	Liu et al. [99]
Tool radius: 5 mm Feed rate: 800 mm/min Form: truncated cone Diameter: 80 mm Height: 25 mm Wall angles: 45°	Continuous DC Manually adjusted	AZ31B magnesium alloy sheets Thickness: 1.4 mm Size: 280 mm × 280 mm	Xu et al. [100]
Tool radius: 5 mm Feed rate: 600 mm/min Step size: 0.1 mm Form: double curvature cup Height: 20 mm Wall angles: 20°, 50°	Continuous DC Current Intensity: 40–120 A	Ti-6Al-4V sheets Thickness: 0.5 mm Size: 320 mm × 320 mm	Valoppi et al. [101]
Tool diameter: 8 mm Feed rate: 1000 mm/min Step size: 0.2 mm Form: squared cone Height: 30 mm Base: 100 × 100 mm Wall angles: 45°		TC4 Ti alloy sheets Thickness: 1 mm Size: 150 mm × 150 mm	Li et al. [102]
Tool diameter: 10 mm Feed rate: 900 mm/min (for all sheet materials) Vertical pitch: 0.3 mm (for Ti-6Al-4V) 0.2 mm (for both AA6061, DC01)	AC transformer (current: 300–1000 A, voltage: 1–5 V) 450 A (for Ti-6Al-4V) 300 A (for both AA6061, DC01)	Ti-6Al-4V, AA6061, DC01 sheets Thickness: 1 mm Size: 100 mm × 100 mm	Vahdani et al. [103]
Tool radius: 8 mm (experiment), 5 mm (analytical model) Interlayer spacing: 2.5 mm Form: hyperbolic truncated cone and hyperbolic truncated pyramid Maximum forming height: 50 mm Maximum forming angle: 90°	Pulsed DC Current densities: 10.1–15.6 A/mm^2^ (for hyperbolic truncated cone) 9.8–15.6 A/mm^2^ (for hyperbolic truncated pyramid)	2024-T3 aluminum alloy sheet	Gao et al. [35]

A few studies considered the effects of various process parameters on the formability of sheets through the application of electric current in EA incremental forming. Honarpisheh et al. [97] carried out an experimental study of EA incremental forming of Ti-6Al-4V sheet apprised with FEM modelling to investigate the effect of various process parameters on the formability of the sheet. The study demonstrated that increasing wall angle, tool diameter, and step size significantly impacted formability, forming force, and thickness reduction. Specifically, at a wall angle of 56° and a tool diameter of 8 mm, increasing the step size from 0.1 mm to 0.3 and 0.5 mm raised the forming force by 37.8% and 47%, respectively. The wall thickness reduction also increased with wall angle, reaching 41.3%, 49.6%, and 74.4% at wall angles of 45°, 56°, and 64°, respectively. It was concluded that with appropriate control of current, feed rate, and step size, high-quality parts with wall angles exceeding 45° can be successfully formed using the EA process.

The significant influence of the radius of the forming tool and the wall angle of the sheet specimen on the temperature control through the electrical current was also reported by Min et al. [98] in a single-point EA incremental forming. The study found that the tool radius and sheet wall angle significantly influence temperature control. These conclusions were drawn from a parametric study using an analytical thermal model, validated through two isothermal experiments conducted at 873 K on CP-K 60/78 and DP1000 high-strength steels. The results highlight the critical importance of optimizing tool geometry to enhance energy efficiency. Based on these insights, real-time temperature monitoring and adaptive correction of material parameters could further improve thermal control accuracy and support the development of intelligent, feedforward control strategies.

Researchers were also focused on addressing the issues associated with geometrical accuracy, surface finish of the formed parts, and tool wear in EA incremental forming of various difficult-to-form alloy sheets. To improve the surface quality and reduce excessive tool wear in EA incremental forming, Liu et al. [99] designed four different forming tools, such as a conventional rigid tool with and without inner water cooling, a rolling-ball tool, and a rolling-wheel tool; their performances were assessed in the forming of Ti-6Al-4V sheet. Based on the experimental results, they conclude that both the rolling-ball tool and rolling-wheel tool provided the best results for EA incremental forming of a Ti-6Al-4V sheet in terms of reducing the surface roughness of the formed part and wear of the tool tip. The tool temperature could be dropped to 100 °C from 500 °C with inner water cooling. Another important observation was that for titanium alloy, a lower current of the order of 200–300 A is adequate for forming than that required for forming aluminum and magnesium alloys. In a similar flavor, Xu et al. [100] also explored the use of different forming tools, such as a rigid tool and roller-ball tool, to enhance the surface finish and geometrical accuracy in the processing of magnesium AZ31B sheets by an improved EA double-sided incremental forming and compared the results with EA single point incremental forming. It was observed that EA double-sided incremental forming provides better geometrical accuracy and surface finish than EA single-point incremental forming. Contrary to the observations made by Liu et al. [99] in the EA incremental forming of titanium alloy, the rigid tool was reported to achieve a better surface finish than the roller-ball tool in the EA double-sided incremental forming of magnesium alloy.

Two forms of EA incremental forming methodologies, namely, EA mixed double-sided incremental forming (E-MDSIF) and EA accumulative double-sided incremental forming (E-ADSIF), were proposed by Valoppi et al. [101] and demonstrated using Ti-6Al-4V. Experimental results showed that E-MDSIF provided superior performance over E-ADSIF, particularly in reducing forming forces. A maximum force reduction of 14.5% was achieved at a current intensity of 100 A, while a higher intensity of 120 A led to only an 8% reduction, likely due to increased tool wear and material oxidation at elevated temperatures. In addition to lowering forming forces, E-MDSIF also improved the geometric accuracy of the formed parts when compared to E-ADSIF. Furthermore, E-MDSIF increased both the hardness and surface roughness of the components without altering their microstructure. These improvements in surface quality and dimensional precision suggest the potential of E-MDSIF for applications in biomedical component manufacturing. To circumvent certain defects associated with the local electric hot incremental forming (LEHIF) process, such as inhomogeneous temperature distribution, arc burns for the sheet and the tool, an integral electric heating design was proposed by Li et al. [102] to form a TC4 Ti alloy sheet. The proposed design directly made the current flow from one side of the sheet into the other side of the sheet, providing a homogeneous temperature distribution in the forming zone. They carried out numerical simulations and experiments using different current values and suggested that a current value of 500 A should be adopted during the forming process using the proposed heating method. They report better geometric accuracy, fewer fracture defects, smoother surface finish, and better forming tool life than LEHIF.

The improvements in formability of Ti-6Al-4V titanium, AA6061 aluminum, and DC01 steel sheets formed by EA hot incremental forming were experimentally investigated by Vahdani et al. [103]. They studied the effect of different lubricants, feed rates, vertical pitches, and electric currents on the fracture depth of the formed parts as a measure of formability using Taguchi design of experiments and analysis of variance methods. They reported graphite as the most effective lubricant and an optimal feed rate of 900 mm/min to achieve the maximum forming depth for all the sheets. The best currents providing the maximum forming depth were reported as 450 A for titanium alloy and 300 A for aluminum and steel alloy sheets. Compared to conventional cold single-point incremental forming (SPIF), EHISF significantly increased the forming depth of Ti-6Al-4V from 3.25 to 35 mm and improved that of AA6061 by 21%. For DC01, no increase in forming depth was observed due to geometric design constraints. Under optimal parameters, surface roughness values were reported as 4.52 µm for Ti-6Al-4V, 1.12 µm for AA6061, and 1.75 µm for DC01, with a corresponding tool wear of 0.12 mm, 0.14 mm, and 0.07 mm, respectively. Furthermore, EHISF improved the wall thickness distribution of the DC01 parts by 18% compared to cold SPIF.

Gao et al. [35] studied the effect of pulsed current on the fracture limit of 2024-T3 aluminum alloy processed by the EA double-side multi-point incremental sheet forming (E-DMISF) method. The study focused on forming hyperbolic truncated cones and pyramids, developing a quantitative fracture criterion based on stress triaxiality as the characteristic integral function, supported by numerical simulations and experimental validation. Results showed that tool compression combined with pulsed current reduced stress triaxiality, increasing the fracture equivalent strains to 0.68001 under plane strain and 0.87323 under biaxial strain. The corresponding fracture integral values of 0.1753 and 0.1798 exceeded the critical threshold of 0.1391, indicating a delayed fracture onset compared to conventional SPIF. The optimal current density for the 2024-T3 aluminum alloy was reported as 15.6 A/mm^2^.

### EA miscellaneous processes

Apart from the machining and metal forming processes, the application of electric current has been investigated in a few other manufacturing processes, for example, punching and blanking of sheets and surface rolling, by a few researchers. These processes are reviewed in this section.

#### EA blanking and punching

**Fig. 7:**
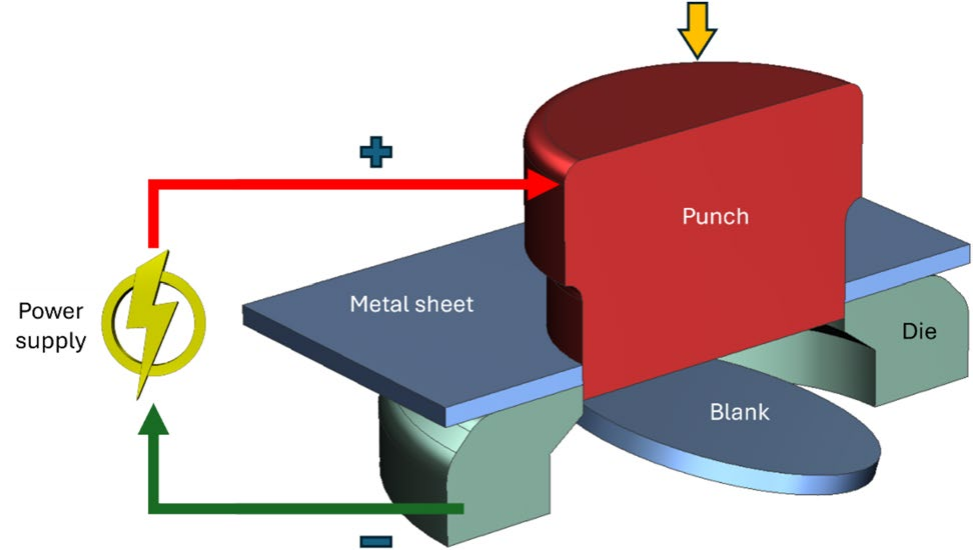
Schematic of EA blanking/punching

In EA blanking, the electric current is passed through the sheet material while the blank is punched out or trimmed. A typical EA blanking/punching process is schematically shown in Fig. 7. The EA blanking of ultra-high strength steels using tensile electroplasticity under a single pulse electric current was experimentally studied by Kim et al. [104]. Three different electric current densities (60, 75, and 90 A/mm^2^) for a constant duration were applied during EA blanking of the sheet to measure the blanking load, and the corresponding results were compared with blanking assisted by local resistance heating (RH blanking). The experimental results demonstrated the decrease of blanking load in EA blanking as compared to RH blanking with the increase of applied current density. A significant reduction of 85% in EA blanking load was reported when compared with the cold blanking. The difference in blanking load between EA blanking and RH blanking was nearly constant for the same electric current densities. A slight increase in burnished surface height was observed in comparison with cold blanking.

Moreover, researchers have sought to develop EA punching methods to reduce the punching load and other disadvantages associated with the conventional punching of sheets. To this end, Yiu et al. [105] developed an experimental setup for EA punching of Fe_78_B_13_Si_9_ metallic glass ribbon with the application of in situ electropulses in different combinations of frequency and punching speed. They punched the ribbon with reduced punching force, aided by electropulsing without inducing shear bands. It was reported that the higher the pulsing frequency, the lower the force needed to punch. The force reduction was observed to be more prominent at higher punching speeds. The processing temperature was reported to be far below the glass transition temperature of the glass ribbon, with a time taken of only about 30 s. Further, X-ray diffraction results showed that post-processing, all the ribbons treated at 14.3 Hz remained amorphous and ribbons treated at 16.8 Hz suffered stress relaxation, retaining crystallinity (also possible embrittlement indicated by sharpening of their halo peak).

The EA punching of 2024T4 aluminum alloy sheet was experimentally investigated by Tang et al. [106, 107] to study the effect of electropulsing on punching load, edge stretchability, and corrosion resistance of the punched part. Tang et al. [106] reported that electropulsing had a significant effect on reducing the punching load and improving the surface quality of the punched profile. At a current density of 100 A/mm^2^, the maximum punching load was reduced by 15.68% when electropulsing was applied prior to punching, and by 21.49% when applied during punching, in comparison to conventional punching methods. Furthermore, the burnish zone of the punched edge increased by up to 86% in the case of punching during the application of electropulsing, indicating a substantial improvement in surface finish. The study also observed significant improvements in the ultimate tensile strength of punched specimens due to reduced edge strain hardening and the healing of microcracks. Tang et al. [107] demonstrated that applying electropulsing at a current density of 100 A/mm^2^ post-punching significantly enhances both the edge stretchability and corrosion resistance of punched parts. These improvements are primarily attributed to localized grain refinement, healing of microcracks, a reduction in dislocation density, and the formation of protective passivation films near the punched edge. Furthermore, electropulsing after punching was shown to effectively reduce deformation forces and increase deformation ratios. These benefits were observed to be more pronounced at higher current densities. A summary of the different process parameters used in the various EA blanking and punching studies, reviewed in this section, is presented in Table 11.

**Table 11:** EA blanking and punching

Process	Current	Specimen	Reference
Blanking Servo press: 1500 kN Punch diameter: 10 mm Punch speed: 12 mm/s Blanking time 100 ms	Constant DC Current density: 60, 75, 90 A/mm^2^ Pulse width: 0.4 s Frequency: 0.0338 Hz	Complex phase (CP) steel sheet Width: 20 mm Thickness: 1.2 mm	Kim et al. [104]
Punching Pneumatic press Punch diameter: 1 mm Punch speed: 3, 7, 10, 15 mm/min Total displacement: 3 mm	Pulsed DC Voltage: 42 V Current: 1 A Pulse width: 0.1 ms Frequencies: 14.3, 16.8 Hz	Metallic glass foil ribbons Length: 80 mm Width: 10 mm Thickness: 25 μm	Yiu et al. [105]
Punching Universal testing machine (5 tons) Punch diameter: 100.9 mm Punch speed: 300 mm/min	Pulsed DC Current density: 50, 75, 100 A/mm^2^ Pulse width: 250 ms Frequency: 1 Hz	2024T4 aluminum alloy sheet Thickness: 1 mm Width: 30 mm Length: 40 mm	Tang et al. [106] Tang et al. [107]

#### EA ultrasonic surface rolling

In the ultrasonic surface rolling process, an ultra-hard processing tip is employed under a static force to roll the material surface while simultaneously a dynamic impact is applied to the surface through the tip by an ultrasonic apparatus in the normal direction to induce severe plastic deformation in the metal surface. When this process is assisted with electropulsing, the process is called EA ultrasonic surface rolling. It has been recognized that both the thermal and athermal effects of electropulsing in EA ultrasonic surface rolling have favorable outcomes on the processing of metal surfaces.

Wang et al. [108–110] experimentally investigated the effect of electropulsing in EA ultrasonic surface rolling of AISI 304 stainless steel. It was reported that compared to the ultrasonic surface rolling without an electric field, EA ultrasonic surface rolling induced healing of surface microcracks, better surface roughness, higher surface microhardness and greater impact depth of the strengthened layer by suitably selecting the pulsed current parameters. In [109, 110], the electropulsing-induced surface residual stresses in the EA surface rolled AISI 304 samples were quantified using the blind hole drilling method and instrumented indentation test, respectively. It was reported that a significantly higher magnitude of compressive residual stresses was generated on the surface at a suitable pulse frequency of 600 Hz, compared to the non-EA ultrasonic surface rolling. For instance, in the specimen treated by EA ultrasonic surface rolling at 600 Hz, the maximum residual compressive stress obtained was −1414 MPa, significantly higher than −943 MPa obtained in conventional ultrasonic surface rolling [110]. The authors also reported improvements in surface wear resistance at 600 Hz. Additionally, in [110], a model describing the evolution of fatigue performance of AISI 304 stainless steel induced by EA ultrasonic surface rolling was proposed based on the experimental results. Wang et al. [111] carried out a similar study for Ti-6Al-4V alloy and reported analogous observations. The suitable electric pulse frequency for the alloy was 250 Hz.

Gradient nanocrystallization and improvements in surface mechanical properties of commercially pure titanium using electropulsing-assisted (EA) ultrasonic surface rolling were experimentally studied by Ye et al. [44]. An optimal frequency of 500 Hz was identified, at which EA ultrasonic surface rolling demonstrated significant advantages over the conventional process. Compared to the conventional ultrasonic surface rolling process, EA ultrasonic surface rolling reduced the maximum wear scar depth to two-thirds and increased surface hardness to 308 HV, representing a 46.7% improvement over the turning sample. Furthermore, the EA process produced a deeper plastic deformation layer of 480 μm, approximately 50% greater than the conventional method, and a lower surface roughness of 0.026 μm. These enhancements are attributed to the combined effects of electropulsing-induced ductility and ultrasonic work hardening, which promote dynamic recrystallization through increased dislocation mobility and density. Additionally, residual stress measurements using the hole drilling method revealed that EA ultrasonic surface rolling induces significantly higher compressive residual stresses compared to conventional ultrasonic surface rolling.

Xie et al. [45] investigated EA ultrasonic surface rolling on CrMnFeCoNi high-entropy alloy, achieving a gradient nanocrystalline structure. Electropulsing increased the yield strength from 356 to 750 MPa and the tensile strength from 519 to 802 MPa, while elongation improved to 21.9% compared to 14.9% achieved with conventional ultrasonic surface rolling. The conventional process produced closely spaced nano-twin boundaries, whereas the electropulsing-assisted method generated dislocation boundaries with spacing ranging from tens to hundreds of nanometers. This structural change enhanced the overall strength-ductility balance. Table 12 summarizes the various process parameters used in the preceding studies on EA ultrasonic surface rolling.

**Table 12:** EA ultrasonic surface rolling

Process parameters	Current	Specimen	Reference
Rolling speed: 24 m/min Feeding rate: 0.1 mm/rev Static force: 700 N Ultrasonic vibration: 8 μm at 30 kHz	Pulsed DC Current density (RMS): 0.62–1.34 A/mm^2^ Pulse width: 60 μs Frequency: 600 Hz	Austenitic stainless steel 304 rods Diameter: 16.8 mm Length: 150 mm	Wang et al. [108]
Distance between electrodes: 80 mm Roller diameter: 14 mm Rolling speed: 24 m/min Feeding rate: 0.1 mm/rev Static force: 700 N Ultrasonic vibration: 8 μm at 30 kHz	Pulsed DC Current density (RMS): 0.78–1.27 A/mm^2^ Pulse width: 60 μs Frequency: 500–700 Hz	Austenitic stainless steel 304 rods Diameter: 16.8 mm Length: 150 mm	Wang et al. [109]
Rolling speed: 24 m/min Feeding rate: 0.1 mm/rev Static force: 950 N Ultrasonic vibration: 8 μm at 30 kHz	Pulsed DC Current density: 6.59, 7.72, 8.85 A/mm^2^ Pulse width: 60 μs Frequency: 550, 600, 650 Hz	AISI 304 stainless steel rods Diameter: 16.8 mm Length: 150 mm	Wang et al. [110]
Distance between electrodes: 80 mm Roller diameter: 14 mm Rolling speed: 10 m/min Feeding rate: 0.05 mm/rev Static force: 1150 N Ultrasonic vibration: 8 μm at 30 kHz	Pulsed DC Current density (RMS): 0.78–1.27 A/mm^2^ Pulse width: 60 μs Frequency: 200–350 Hz	Titanium alloy Ti-6Al-4V Diameter: 13.6 mm Length: 150 mm	Wang et al. [111]
Distance between electrodes: 120 mm Roller diameter: 28 mm Rolling speed: 12.1 m/min Feeding rate: 13 mm/min Static force: 1030 N Ultrasonic vibration: 6 μm at 27 kHz	Pulsed DC Current density (RMS): 0.608–1.028 A/mm^2^ Frequency: 400–600 Hz Pulse width: 135 μs	Commercial pure titanium bar Diameter: 14.8 mm Length: 150 mm	Ye et al. [44]
Roller diameter: 14 mm Rolling speed: 15 m/min Feeding rate: 15 mm/min Static force: 800 N Ultrasonic vibration: 6 μm at 30 kHz	Pulsed DC Current density: 30 A/mm^2^ Frequency: 300 Hz Pulse width: 70 μs	Cr-Mn-Fe-Co–Ni alloy	Xie et al. [45]

## Trends and challenges

A comprehensive review of the various EA manufacturing processes has been presented here, which demonstrates that EA manufacturing has several advantages over and above conventional manufacturing processes, including, in some cases, hybrid manufacturing techniques. A common observation is that the pulsed vs continuous EA yield a varied response, and as such, implies that the nature of the current form plays a crucial role in the manufacturing outcomes.

We make the following observations and challenges in EA manufacturing processes. These are:

- A lack of clear understanding of the interplay between electroplasticity and Joule heating. It is clear that pulsed direct current demonstrates, in most cases, a clear advantage when compared to continuous currents. However, the exact nature of the current waveform and its implications for the machining outcomes need further research.
- Machine tools need to be developed for appropriate EA manufacturing applications. A crucial point which could potentially affect tool life is the effect of electroplasticity on the tool materials. It may be surmised that the very effects of pulsed current could potentially self-soften the machine tools. Some research shows that carbide tools do not suffer from this effect; however, further research is needed, and there is also a need for tool development for commercial viability.
- Studies in EA manufacturing must incorporate a thorough assessment of carbon costs for manufacturing sustainability. The application of pulsed current will incorporate the addition of more energy costs in the machining process. The overall benefit from EA manufacturing must outweigh all forms of conventional manufacturing, which is the current norm. This must be performed without bias, including the assessment of tool wear and structural integrity of manufactured parts.
- With limited studies available, EA milling remains a largely unexplored process offering significant potential for future research.

We also make an overall observation that all conventional/traditional manufacturing processes have had the advantage of decades of research, which incorporate various optimization strategies for efficient manufacture. Tool development has also happened in parallel to the effect that most conventional manufacturing processes incorporate optimized machine tools with optimized manufacturing systems. However, EA manufacturing still lacks this foundational development. A key challenge in EA manufacturing is optimizing the complex interplay of electrical, thermal, and mechanical parameters. Traditional optimization methods often fall short, but modern metaheuristic algorithms, such as equilibrium optimizer [112, 113], golden jackal optimization [114, 115], and quasi-oppositional equilibrium algorithm [116], show promise for enhancing optimization and control of multiple interdependent parameters governing EA processes. For EA manufacturing to be industrially viable, sustained efforts are required not only in advanced algorithm-driven process optimization but also in the design, development and manufacture of standardized, dedicated machine tools. However, such progress has been slow, and EA manufacturing has seen limited industrial adoption. This is primarily due to the lack of process standardization, complex equipment setup involving safety and control challenges, and a limited understanding of electroplastic effects across different materials and scales. Nevertheless, industry has begun to explore the potential of electrically driven forming processes. For instance, Ford Motor Company’s collaboration with Pacific Northwest National Laboratory on electrohydraulic forming (EHF) for prototyping near-net-shape automotive panels [117]. A related patent by Ford Global Technologies, LLC can also be found [118]. Although EHF uses high-voltage discharge to generate shockwaves, a mechanism that differs from conventional current-assisted methods, it demonstrates the practical industrial application of electrical energy in metal forming. This example highlights the growing interest in applying electrical assistance to improve efficiency and precision in EA manufacturing in general.
